# Desaturation during Six-Minute Walk Testing Predicts Major Morbidity Following Anatomic Lung Resection among Patients with COPD

**DOI:** 10.3390/healthcare7010016

**Published:** 2019-01-23

**Authors:** Christopher W. Towe, Katherine Wu, Alina Khil, Yaron Perry, Stephanie G. Worrell, Vanessa P. Ho, Philip A. Linden

**Affiliations:** 1 Division of Thoracic and Esophageal Surgery, Department of Surgery, University Hospitals Cleveland Medical Center, Case Western Reserve School of Medicine, 11100 Euclid Avenue, Cleveland, OH 44106, USA; kmw115@case.edu (K.W.); axk938@case.edu (A.K.); yaron.perry@uhhospitals.org (Y.P.); Stephanie.Worrell@UHhospitals.org (S.G.W.); philip.linden@uhhospitals.org (P.A.L.); 2 Division of Trauma, Critical Care, Burns, and Emergency General Surgery, MetroHealth Medical Center, Case Western Reserve School of Medicine, Cleveland, OH 44109, USA; vho@metrohealth.org

**Keywords:** thoracic surgery, pulmonary function testing, six-minute walk, surgery, risk-stratification

## Abstract

Background: Pulmonary function testing (PFT) is commonly used to risk-stratify patients prior to lung resection. Guidelines recommend that patients with reduced lung function, due to chronic lung conditions such as Chronic Obstructive Pulmonary Disease (COPD), should receive additional physiologic testing to determine fitness for resection. We reviewed our experience with six-minute walk testing (SMWT) to determine the association of test results and post-operative complications. Methods: Consecutive adult patients undergoing segmentectomy, lobectomy, bilobectomy or pneumonectomy between 1 January, 2007 and 1 January, 2017 were identified in a prospectively maintained database. Patients with poor lung function, as defined by percent predicted forced expiratory volume in 1 s (FEV1) or diffusion capacity of carbon monoxide (DLCO) ≤60%, had results of SMWT extracted from their chart. Association of test result to post-operative events was performed. Results: 581 patients had anatomic lung resections with predicted post-operative FEV1 or DLCO values ≤60%, consistent with a diagnosis of COPD. Among them, 50 (8.6%) had preoperative SMWT performed. Patients who received SMWT were more likely to have a FEV1 or DLCO less than 40 percent predicted (24/50 (48.0%) vs 166/531 (31.3%), *p* = 0.016). Post-operatively, patients who had SMWT performed had higher rates of pneumonia, but similar rates of major morbidity. The post-exercise oxygen saturation and the amount of desaturation correlated with the occurrence of major morbidity. In multivariable regression, oxygen desaturation was an independent risk factor for the occurrence of major morbidity, and desaturation was an excellent predictor of major morbidity by receiver operating characteristic curves analsysis. Conclusions: Among patients with elevated risk, oxygen desaturation during SMWT was independently associated with the occurence of major morbidity in multivariable analysis, while pulmonary function testing was not. SMWT is an important tool for risk-stratification, and may be underutilized.

## 1. Background

Assessing the ability of patients to tolerate anatomic lung resection is complex. Patient selection for anatomic lung resection is based on a multifactorial assessment. Presently, the American College of Chest Physicians (ACCP) provides guidelines for the preoperative assessment of lung function prior to anatomic lung resection [1]. Due to the high prevalence of Chronic Obstructive Pulmonary Disease (COPD) in patients undergoing lung cancer resection, evaluation of resectability begins with pulmonary function tests (PFTs). PFTs are used to obtain a forced expiratory volume in one second (FEV1) and the diffusion capacity of carbon monoxide (DLCO), and these values are adjusted to a percent predicted post-operative (pppo) value based on the extent of resection performed. To summarize the ACCP recommendations, patients with pppoFEV1 and pppoDLCO values greater than 60% expected should tolerate resections including pneumonectomy (Grade 1C) [1]. In patients with intermediate expected DLCO or FEV1 (pppo values between 30 and 60%), further characterization of lung function by stair climbing or walk testing is recommended (Grade 1C) [1]. Patients with pppoFEV1 and pppoDLCO values less than 30% of expected should undergo cardiopulmonary exercise testing to further stratify the ability to tolerate resection (Grade 1B) [1]. Recent studies however have questioned the relationship of PFTs to perioperative risk, especially in the setting of thoracoscopic surgery [2,3,4,5]. These categories are similar to the Global Initiative for Chronic Obstructive Lung Disease (GOLD) classification of COPD, which defines moderate COPD as FEV1 between 50% and 80%, severe COPD as FEV1 between 30% to 50%, and very severe COPD as FEV1 less than 30%. ACCP guidelines are vague regarding high risk patients, and suggest that “surgery can be performed safely in selected patients with markedly abnormal lung function at baseline” without offering criteria for surgical selection [1].

Currently, stair climbing and walking tests are adjunct tests for intermediate risk patients. As a surrogate for formal cardiopulmonary exercise testing (CPET), these tests are simple, rapid, and economical to perform. The ability to climb three flights of stairs effectively identifies patients who are at low risk of post-operative complications following lobectomy [6,7]. The six-minute walk test (SMWT) and the shuttle walk test are other physiologic tests used to assess cardiopulmonary function prior to surgery [8]. Studies suggest that there is a relationship between SMWT walk distance and CPET, but there is no standardized method to discriminate patients at higher post-operative risk based on test results [8]. Oxygen desaturation during exercise has been proposed as a way to identify high risk patients but was not associated with increased risk of post-operative cardiopulmonary complications [9]. We believe that this study was underpowered to detect a difference because it was applied to all patients receiving lung resection. Another study, which was also not performed in a high risk cohort, demonstrated that SMWT distance walked <450 m was a threshold for predicting pneumonia with 69.2% sensitivity and 71.1% specificity, and distance walked <450 m was an independent risk factor for pneumonia in a logistic model [10]. The generalizability of these findings are questionable given the cohorts used and the lack of comparison to standard lung function measurements.

The utility of walking tests in the assessment of patients undergoing thoracic surgery is currently unclear. While the ACCP continues to recommend walking tests as part of their “Algorithm for Thoracotomy and Major Anatomic Lung Resection” [1], these tests have not been appropriately validated in the setting of contemporary perioperative care and thoracoscopic surgery. We hypothesized that the six-minute walk test would identify patients at highest risk of major morbidity after anatomic lung resection among patients with COPD and significantly reduced lung function.

## 2. Methods

Consecutive patients undergoing anatomic lung resection between 1 January, 2007 and 1 January, 2017 by the Division of Thoracic and Esophageal Surgery at the University Hospital Cleveland Medical Center were identified in our prospectively maintained database. All adult patients undergoing segmentectomy, lobectomy, bilobectomy or pneumonectomy were included. The hospital chart of each patient was reviewed to define the surgical procedure performed, preoperative lung function test result, preoperative SMWT result, and post-operative complications. Percent predicted post-operative (pppo) values for PFTs assumed that there were 19 segments of the lung, and were calculated using standard technique [1]. Unless otherwise noted, patients were assumed to have 19 functioning segments at the start of the cohort. We utilized the standard Society of Thoracic Surgeons (STS) definition for procedures performed and post-operative complications [11,12]. In order to include only moderate risk and high risk patients, we included only patients whose pppo values for FEV1 or DLCO were less than 60%, as per the current AACP recommendations for preoperative assessment of patients undergoing surgery for lung cancer [1].

Development of major morbidity was the primary outcome assessed in this study, as we believe the risk of these complications may alter surgical decision making. A complication was defined by the occurrence of any adverse outcome as defined by the STS and was not graded by severity [11]. Major morbidity was defined as patients with: tracheostomy, reintubation, initial ventilator support greater than 48 h, adult respiratory distress syndrome, bronchopleural fistula, pulmonary embolus, pneumonia, bleeding requiring reoperation, myocardial infarction, or death within 30 days of the procedure [11].

The decision to perform SMWT was based on surgeon preference, and results were included for analysis if the test occurred within 365 days of surgery. SMWT was performed on a flat surface in a straight corridor under direct supervision based on published guidelines [8]. Test results included oxygen saturation, heart rate, and dyspnea score at the beginning and end of exercise. Desaturation was defined as the change in oxygen saturation (pre-walk minus post-walk), such that the value represents how much lower a patient’s oxygen saturation was after the test (relative to prior). Dyspnea score was defined using the Borg Scale [8,13]. Other data collected included distance walked and the number of times the patient stopped or rested during the walk. The distance walked in 6 min was compared to a “predicted lower limit” as an absolute value and as a percent predicted distance. The calculated value “percent predicted distance” was defined by the actual distance walked divided by the predicted value, and multiplied by 100.

Data were evaluated for normality using the “skewness/kurtosis” method as well as the Shapiro-Wilk test. Continuous variables were presented as median and interquartile range. Two group comparisons were performed using Student’s *t*-test or Wilcoxon rank-sum for continuous variables (such as age, heart rate or desaturation) and chi-square or Fisher’s Exact analysis for categorical variables (such as sex) as appropriate (α < 0.05). Receiver operating characteristic (ROC) analysis was performed to determine the ability of various test results to discriminate the outcome of major morbidity. For ROC analysis, data were presented as Area Under the Curve (AUC or c-statistic); this statistic value ranges from 0 to 1, with 0.5 indicating that the model performs no better than chance, and values of 0.7 or higher are generally accepted as a “strong” model [14]. Discriminatory values from the ROC analysis were chosen based on highest “accuracy”. We also performed multivariable regression using covariates selected from the univariate analysis using backwards selection (α < 0.05). All data were analyzed using STATA/IC, Version 14.2 (StataCorp, College Station, TX). The study was approved by the hospital’s Institutional Review Board.

## 3. Results

During the study period, 922 patients underwent anatomic lung resection. Within this cohort, 581 patients had pppoFEV1 or pppoDLCO values less ≤60%. Among the 581 patients with pppoFEV1 or pppoDLCO values less ≤60%, 50 (8.6%) had preoperative SMWT performed. The charcteristics of this cohort and a comparison of patients with and without SMWT performed is shown in Table 1. Patients with SMWT tended to have higher rates of coronary artery disease and lower pulmonary function test results. Patients who received SMWT were also more likely to have a FEV1 or DLCO less than 40 pecent predicted (24/50 (48.0%) vs. 166/531 (31.3%), *p* = 0.016). Post-operatively, patients who had SMWT performed had similar rates of overall and major morbidity.

Among the 50 patients who performed SMWT, the relationship of test results to post-operative major morbidity is shown in Table 2. The post-exercise oxygen saturation and the amount of desaturation correlated with the occurrence of major morbidity, but not the occurrence of any complication. Post-exercise heart rate, relative change in heart rate, Borg score, distance walked, and percent predicted distance were not associated with the occurrence of any complication or major morbidity. Walking less than the predicted distance was not associated with the occurrence of any complication (*p* = 0.43) or major complication (*p* = 0.35). Pulmonary function test results were also related to occurrence of post-operative major morbidity. Both DLCO and FEV1 were associated with the occurrence of any complication, while FEV1 alone was associated with major morbidity.

**Figure 1:**
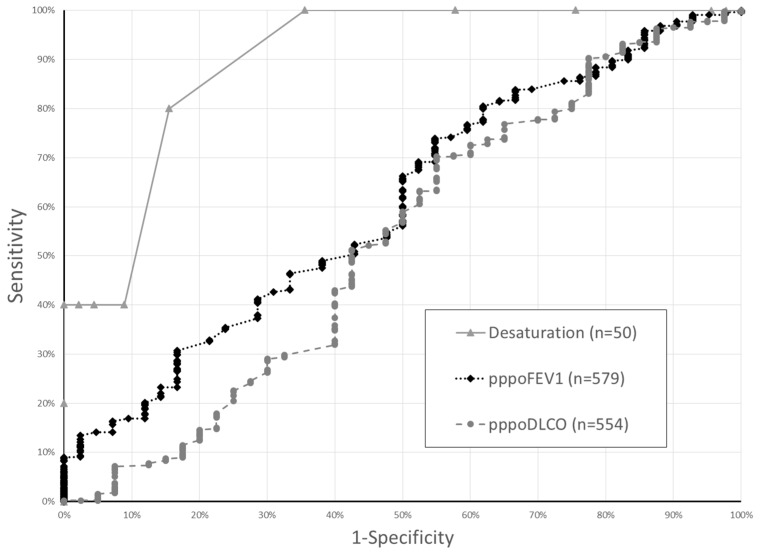
Receiver operating characteristic curves for amount of desaturation during six-minute walk and pulmonary function test results to detect major morbidity among 581 patients with pppoFEV1 or pppoDLCO values less ≤60%. For a given test, each data point represents a result of that test and its associated sensitivity and specificity. The area under the curve is a measure of test accuracy. (Abbreviations: pppoFEV1: percent predicted post-operatieve forced expiratory volume in 1 s, pppoDLCO: percent predicted post-operative diffusion capacity of carbon monoxide).

To determine the association of SMWT with the occurrence of major morbidity relative to other pulmonary function testing, we performed ROC analysis. ROC analysis suggested that desaturation was an excellent predictor of major morbidty (c-statistic: 0.90, std error 0.05). Using a cutpoint value of 10, desaturation during SMWT had a sensitivity of 40%, specificity of 100%, and correctly classified 94% of patients. By comparison, ROC analysis of pppoFEV1 and pppoDLCO showed only “fair” or “poor” discrimination of major morbidity in this cohort. (pppoFEV1: 0.60 (0.51–0.69); pppoDLCO: 0.53 (0.42–0.64)) (Figure 1) Among the cohort of all PFT results, using a cutpoint value of 30.2, pppoFEV1 had a sensitivity of 96.7%, specificity of 11.9%, and correctly classified 90.5% of patients. For pppoDLCO, cutpoint values of 27.3 had a sensitivity of 96.3%, specificity of 12.5%, and correctly classified 90.3% of patients. In the cohort of patients with both ppoFEV1 and SMWT, there was a trend towards improved discrimination of major morbidity by desaturation relative to pppoFEV1 (0.900 vs. 0.649, *p* = 0.116).

We also performed a multivariable regression. Variables included in the regression included SMWT desaturation and pppoFEV1, as these were the only factors associated with major morbidity in univariate testing. The regression evaluated the relationship of oxygen desaturation during SMWT and pppoFEV1 to the occurrence of major morbidity (Table 3). This analysis demonstrated that a decrease in oxygen saturation after exercise (desaturation) was associated with major morbidity (OR 1.53:1.03–2.28, 0.035) independently of pppoFEV1 (OR 1.00:0.91–1.10, 0.943).

## 4. Discussion/Conclusions

Preoperative assessment of patients using walking tests is recommended by guidelines, but how these tests should be used to discriminate whether a patient should receive lung resection are currently unclear. Furthermore, while the ACCP continues to recommend walking distance as part of their “Algorithm for Thoracotomy and Major Anatomic Lung Resection” [1], these tests have not been appropriately validated in the setting of contemporary perioperative care and thoracoscopic surgery.

In this cohort of patients with marginal lung function, oxygen desaturation during SMWT was the only test result (from SMWT) that was associated with the occurrence of major morbidity. ROC analysis also suggested that desaturation was a strong predictor of major morbidity, and desaturation was independently associated with the occurance of major morbidity in multivariable analysis, whilst pulmonary function testing results were not.

Although pulmonary function testing is a common and recommended method for patient selection for surgery, the relationship between PFT results and outcome have been questioned [2,4]. Our data suggests that SMWT is a valuable tool for perioperative risk assessment, in that among these higher risk patients, desaturation during the test was associated with major morbidity in multivariable regression, while FEV1 was not. Our study supports evidence that while pulmonary function test results are related to surgical outcomes, poor PFT results may not be the best method to identify patients at risk for major morbidity. Other studies have demonstrated that the distance walked during SMWT was predictive of post-operative complications [10]. Our data do not show this relationship. The difference may be due to patient selection, as the Hattori study was performed on all patients recieiving lung resection surgery, while these data are a subset of patients with marginal lung function. This dynamic may have also been present in a study which found no association of desaturation and post-operative complications [9].

This study has several limitations. There was no standardized protocol at our institution to determine which patients should receive the physiologic testing, and our rate of testing was low. This implies that there is a selection bias in the administration of the test, which may alter the generizability of these findings. This is corroborated by the fact that patients who received testing had worse pulmonary function test results and increased comorbid conditions. This may affect the generalizability of this test to the larger population of patients with pppoFEV or pppoDLCO 30–60%. As a retrospective study, we also cannot ascertain whether there were patients who were not offered an operation based on results of their SMWT. This selection bias may limit the accuracy of the test results. Furthermore, we concede that other unmeasured variables may have effected the study results. For example, a lower extremity ampuation may significantly alter the relationship of walking distance to physiologic status. Another weakness of the study is that the size of the study cohort who received SMWT may limit the ability to detect a benefit to other test results. Despite these limitations, our data suggests that SMWT, which is an easy and low-cost measure of preoperative physiologic function, may be an underutilized way to accurately risk-stratify patients prior to anatomic lung surgery.

We advocate for the use of SMWT in patients with marginal lung function, as supported by national guidelines. We believe that desaturation during SMWT is preferred to pulmonary function testing among high risk individuals as a method to identify patients at an increased risk of major morbidity. We believe that future research into the appropriate cohort for SMWT and determination of “cutoff” values to discriminate patients at the highest risk of post-operative complications is appropriate.

## Figures and Tables

**Table 1:** Characteristics of patients with pppoFEV1 or pppoDLCO ≤60% subdivided by whether they received six-minute walk testing. Data presented as mean (median:IQR) or N (%). Comparison using two tailed *t*-test or chi squared.

Characteristic		SMWT not Performed (n = 531)	SMWT Performed (n = 50)	*p*-Value
Age		67.2 (67.9:60.5–75.2)	70.0 (70.6:64.2–78.2)	0.21
Male Sex		223 (42.0%)	21 (42.0%)	1.00
Body Mass Index		27.3 (26.7:23.1–30.8)	27.1 (26.7:22.3–30.8)	0.99
Hypertension		322 (60.6%)	28 (56.0%)	0.52
Congestive Heart Failure		23 (4.3%)	5 (10.0%)	0.07
Coronary Artery Disease		97 (18.3%)	16 (32.0%)	0.02
Diabetes		87 (16.4%)	10 (20.0%)	0.51
Preopchemotherapy and/or radiation		50 (9.4%)	5 (10.0%)	0.89
pppoFEV1 *		54.6 (54.5:44.2–62.6)	49.4 (47.1:39.8–58.4)	0.02
COPD Classification *	Mild (FEV1 ≥ 80%)	212 (39.9%)	11 (22.0%)	0.024
	Moderate (80% > FEV1 ≥ 50%)	281 (52.9%)	32 (64.0%)	
	Severe (50% > FEV1 ≥ 30%)	33 (6.2%)	7 (14.0%)	
	Very Severe (FEV1 < 30%)	5 (0.9%)	0	
pppoDLCO		49.7 (50.1:40.5–57.5)	42.8 (43.6:37.6–50.2)	<0.01
Surgery Duration (hours)		3.26 (30.5:2.4–3.9)	3.24 (3.20:2.6–3.9)	0.87
VATS		347 (65.4%)	26 (52.0%)	0.06
Number of segments removed at surgery		5.2 (5:5–5)	5.0 (5:4–5)	0.32
Any complication		156 (29.4%)	19 (38.0%)	0.20
Major morbidity		37 (7.0%)	5 (10.0%)	0.43
Death		10 (1.9%)	1 (2.0%)	0.95

* n = 579 with FEV1 available. Abbreviations: pppoFEV1: percent predicted post-operatieve forced expiratory volume in 1 s, pppoDLCO: percent predicted post-operative diffusion capacity of carbon monoxide, VATS: video assitsed thoracoscopic surgery.

**Table 2:** The relationship of six-minute walk test (SMWT) and pulmonary function testing to perioperative major morbidity among 581 patients with pppoFEV1 or pppoDLCO values less ≤60%.

Test Result	Major Morbidity Occured	No Major Morbidity	*p*-Value
Oxygen saturation before SMWT (n = 50) *	96.4 (1.81)	96.4 (1.91)	1.00
Oxygen saturation after SMWT (n = 50) **	90 (89–92)	94 (93–96)	0.010
SMWT Desaturation (change in SpO2) (n = 50) **	4 (4–10)	2 (1–3)	0.003
Heart rate before SMWT (n = 50) *	93.4 (9.8)	74.4 (11.5)	0.10
Heart rate after SMWT (n = 50) **	113.6 (102–130)	98.7 (87–108)	0.13
Borg Score at end of SMWT (n = 46) *	3.20 (2.39)	2.37 (1.28)	0.22
Distance walked (Meters) (n = 50) *	399.2 (114.0)	340.1 (101.7)	0.23
SMWT distance/predicted distance (n = 50) *	0.98 (0.32)	1.06 (0.34)	0.63
pppoFEV1 (n = 579) **	48.4 (39.3–58.2)	54.6 (44.2–61.9)	0.02
pppoDLCO (n = 554) **	49.2 (37.4–58.2)	49.2 (405–56.7)	0.55

* Data presented as mean (SD). Comparison using two tailed *t*-test. ** Data presented as median (IQR). Comparison using Wilcoxon rank-sum. Abbreviations: pppoFEV1: percent predicted post-operatieve forced expiratory volume in 1 s, pppoDLCO: percent predicted post-operative diffusion capacity of carbon monoxide, SPO2: oxygen pulse oximerty value.

**Table 3:** Multivariable logistic regression of factors associated with postoperative major morbidity (n = 50).

Major Morbidity	OR	95% Confidence Int		Z	*p*-Value
SMWT Desaturation (change in SpO2)	1.56	1.04	2.32	2.17	0.030
pppoFEV1	1.00	0.91	1.10	0.07	0.943

LR chi (3) = 10.26, Prob > chi2 = 0.0059, PseudoR2 = 0.316.

## References

[1] Brunelli A., Kim A.W., Berger K.I., Addrizzo-Harris D.J. (2013). Physiologic evaluation of the patient with lung cancer being considered for resectional surgery: Diagnosis and management of lung cancer, 3rd ed: American College of Chest Physicians evidence-based clinical practice guidelines. Chest J..

[2] Berry M.F., Villamizar-Ortiz N.R., Tong B.C., Burfeind W.R., Harpole D.H., D’Amico D.A., Onaitis D.M.W. (2010). Pulmonary function tests do not predict pulmonary complications after thoracoscopic lobectomy. Ann. Thorac. Surg..

[3] Kachare S., Dexter E.U., Nwogu C., Demmy T.L., Yendamuri S. (2011). Perioperative outcomes of thoracoscopic anatomic resections in patients with limited pulmonary reserve. J. Thorac. Cardiovasc. Surg..

[4] Paul S., Andrews W.G., Nasar A., Port J.L., Lee P.C., Stiles B.M., Altorki N.K. (2013). Outcomes of lobectomy in patients with severely compromised lung function (predicted postoperative diffusing capacity of the lung for carbon monoxide % ≤ 40%). Ann. Am. Thorac. Soc..

[5] Ceppa D.P., Kosinski A.S., Berry M.F., Betty C.D., Harpole D.H., Mitchel M.D., D’Amico M.D., Onaitis M.W. (2012). Thoracoscopic lobectomy has increasing benefit in patients with poor pulmonary function: A Society of Thoracic Surgeons Database analysis. Ann. Surg..

[6] Brunelli A., Al Refai M., Monteverde M., Borri A., Salati M., Fianchini A. (2002). Stair climbing test predicts cardiopulmonary complications after lung resection. Chest J..

[7] Olsen G.N., Bolton J.W., Weiman D.S., Hornung C.A. (1991). Stair climbing as an exercise test to predict the postoperative complications of lung resection. Two years’ experience. Chest J..

[8] (2002). American Toracic Society Statement: Guidelines for the six-minute walk test. Am. J. Respir. Crit. Care Med..

[9] Varela G., Cordovilla R., Jimenez M.F., Novoa N. (2001). Utility of standardized exercise oximetry to predict cardiopulmonary morbidity after lung resection. Eur. J. Cardiothorac. Surg..

[10] Hattori K., Matsuda T., Takagi Y., Nagaya M., Inoue T., Nishida Y., Nasegawa Y., Kawaguchy K., Fukui T., Ozeki N. (2017). Preoperative six-minute walk distance is associated with pneumonia after lung resection. Interac. Cardiovasc. Thorac. Surg..

[11] Kozower B.D., Sheng S., O’Brien S.M., Liptay M.J., Lau C.A., Jones D.R., Shahai D.M., Wright C.D. (2010). STS database risk models: Predictors of mortality and major morbidity for lung cancer resection. Ann. Thorac. Surg..

[12] Shapiro M., Swanson S.J., Wright C.D., Chin C., Sheng S., Wisnivsky J., Weiser T.S. (2010). Predictors of major morbidity and mortality after pneumonectomy utilizing the Society for Thoracic Surgeons General Thoracic Surgery Database. Ann. Thorac. Surg..

[13] Mador M.J., Rodis A., Magalang U.J. (1995). Reproducibility of Borg scale measurements of dyspnea during exercise in patients with COPD. Chest J..

[14] Merkow R.P., Hall B.L., Choen M.E., Dimick J.B., Wang E., Chow W.B., Ko C.J., Bilmoria K.Y. (2012). Relevance of the C-statistic when evaluating risk-adjustment models in surgery. J. Am. College Surg..

